# To be or not to be on: aspirin and coronary artery bypass graft surgery

**DOI:** 10.3389/fcvm.2024.1451337

**Published:** 2024-08-26

**Authors:** Aashray K. Gupta, Joshua G. Kovoor, Alasdair Leslie, Peter Litwin, Brandon Stretton, Ammar Zaka, Pramesh Kovoor, Stephen Bacchi, Jayme S. Bennetts, Guy J. Maddern

**Affiliations:** ^1^ Discipline of Surgery, University of Adelaide, Adelaide, SA, Australia; ^2^ Department of Cardiothoracic Surgery, Royal North Shore Hospital, Sydney, NSW, Australia; ^3^ Australian Safety and Efficacy Register of New Interventional Procedures–Surgical, Royal Australasian College of Surgeons, Adelaide, SA, Australia; ^4^ Department of Medicine, Royal Adelaide Hospital, Adelaide, SA, Australia; ^5^ Department of Medicine, Gold Coast University Hospital, Southport, QLD, Australia; ^6^ Department of Cardiology, Westmead Hospital, Westmead, NSW, Australia; ^7^ School of Medicine, Monash University, Melbourne, VIC, Australia; ^8^ Department of Cardiothoracic Surgery, Victorian Heart Hospital, Melbourne, NSW, Australia; ^9^ Research, Audit and Academic Surgery, Royal Australasian College of Surgeons, Adelaide, SA, Australia

**Keywords:** aspirin, coronary artery bypass graft (CABG) surgery, bleeding, perioperative care, outcomes

## Abstract

Aspirin's role in secondary prevention for patients with known coronary artery disease (CAD) is well established, validated by numerous landmark trials over the past several decades. However, its perioperative use in coronary artery bypass graft (CABG) surgery remains contentious due to the delicate balance between the risks of thrombosis and bleeding. While continuation of aspirin in patients undergoing CABG following acute coronary syndrome is widely supported due to the high risk of re-infarction, the evidence is less definitive for elective CABG procedures. The literature indicates a significant benefit of aspirin in reducing cardiovascular events in CAD patients, yet its impact on perioperative outcomes in CABG surgery is less clear. Some studies suggest increased bleeding risks without substantial improvement in cardiac outcomes. Specific to elective CABG, evidence is mixed, with some data indicating no significant difference in thrombotic or bleeding complications whether aspirin is continued or withheld preoperatively. Advancements in pharmacological therapies and perioperative care have evolved significantly since the initial aspirin trials, raising questions about the contemporary relevance of earlier findings. Individualized patient assessments and the development of risk stratification tools are needed to optimize perioperative aspirin use in CABG surgery. Further research is essential to establish clearer guidelines and improve patient outcomes. The objective of this review is to critically evaluate the existing evidence into the optimal management of perioperative aspirin in elective CABG patients.

## Introduction

The role of aspirin in secondary prevention for patients with known Coronary Artery Disease (CAD) is well established, with the initial landmark trials published nearly 40 years ago today. However, its use peri-operatively is more controversial, where the relative risks of thrombosis and bleeding must be carefully balanced. This is particularly true in cases of coronary artery bypass graft (CABG) surgery, where postoperative complications such as bleeding requiring transfusion, reoperation or saphenous vein graft occlusion leading to re-infarction must be considered (1). Evidence shows clear benefit for continuation of aspirin in patients awaiting CABG following Acute Coronary Syndrome (ACS) where risk of re-infarction outweighs bleeding risk (2, 3). However, the evidence is less clear in the case of elective CABG, with ongoing uncertainty regarding optimum perioperative aspirin management. Current guidelines generally recommend continuation of low-dose aspirin in non-emergent cardiac surgery, yet the evidence remains mixed. A summary of the available evidence is displayed in Table 1.

**Table 1:** Key aspects regarding the use of aspirin in patients undergoing CABG.

Mechanism of action	Irreversibly inhibits COX-1 in platelets, reducing thromboxane A2 formation, and preventing platelet aggregation
Benefits in CAD	Secondary prevention – reduces risk of myocardial infarction, stroke, and other vascular events
Perioperative controversy	Debate over balancing thrombosis prevention and bleeding risks during CABG surgery
Acute coronary syndrome	Continuation of aspirin recommended due to high risk of re-infarction outweighing bleeding risk
Elective CABG	Mixed evidence on the benefits and risks of continuing aspirin preoperatively
Thrombotic risks	Aspirin helps prevent saphenous vein graft occlusion and subsequent myocardial infarction post-CABG
Bleeding risks	Increased risk of major bleeding, transfusion requirements, and reoperation when aspirin is continued
Clinical guidelines	Generally recommend continuation of low-dose aspirin in non-emergent cardiac surgery, though evidence varies
Risk stratification	Lack of specific tools to guide perioperative aspirin use in CABG patients
Individualized care	Emphasis on tailored approaches considering patient-specific thrombotic and bleeding risks

The key evidence for use of aspirin in CAD is summarized in a series of worldwide meta-analyses by the Antiplatelet Treatment Trialists' (ATT) Collaboration, established in the 1990s with the purpose of evaluating the role of antiplatelet agents in cardiovascular disease (CVD). In the 1994 ATT metanalysis, 20,000 patients with a previous Myocardial Infarction (MI) were included, with aspirin shown to significantly reduce the rate of vascular events over a 2-year period (4). A subsequent meta-analysis by the same group in 2002 included 195 randomized trials and 135,000 patients with a history of cardiovascular disease. Antiplatelet therapy, predominantly low dose aspirin, was again shown to reduce the risk of subsequent vascular events by nearly one quarter (5). As a result, aspirin remains a mainstay in current day guidelines for the management of both stable and unstable coronary disease, including patients pre and post CABG, where benefit was also shown (6–8).

However, as aforementioned, the use of aspirin in the perioperative period has long been a source of contention, in both cardiac and non-cardiac surgery. Ultimately the question relates to the relative risks of thrombosis vs. bleeding in an individual patient. Factors to be considered include the indication for aspirin, particularly in cases of coronary stenting, and patient specific bleeding factors. Although recent guidelines suggest class I evidence for low-dose aspirin to be continued preoperatively in patients undergoing non-emergent cardiac surgery, some studies have found this has negligible effect on cardiac outcomes (9). The POISE-2 study, a large US-based trial found that in 10,010 patients undergoing non-cardiac surgery there was a significant increase in the risk of major bleeding with no significant effect on the rate of composite death or non-fatal myocardial infarction (10). It should be noted, however, that only 10% of patients included had a history of CABG or coronary stenting and nearly 40% of procedures were orthopaedic, limiting the application of the findings to cardiac surgery patients.

In order to address this relative dearth of cardiac surgery specific evidence, the Australian ATACAS study published in 2016 examined 5,784 patients undergoing elective CABG, of which 2,100 were randomized to receive either aspirin or placebo preoperatively. The study concluded that preoperative aspirin did not result in lower thrombotic complications or higher bleeding complications (11). This was bookended by meta-analyses in 2015 and 2017, which both showed reduced rates of peri-operative MI and increased post-operative bleeding in the aspirin arms, though no mortality benefit (12, 13). However, both cited the significant heterogeneity of studies and relative lack of high-quality randomized studies in their discussions. As a result, their applicability to individual patients remains poor. Should aspirin in fact be held preoperatively in certain patient groups, and if so, for how long? And if held, how soon postoperatively should it be recommenced? These questions remain unanswered, including a means of assessing which patients will derive greatest benefit from continuation perioperatively. Whilst there do exist validated risk scoring systems for CABG, such as the STS score, along with mortality risk calculators, such as the Euroscore and AUSscore II model, they do not address nor provide guidance for the perioperative use of aspirin.

To further confound the picture, modern medication regimens have changed since the initial evidence base for aspirin in CAD was established nearly 40 years ago. Only two of the aforementioned trials even recorded beta blocker or statin use in their patients, with both being prescribed in less than 20% of patients with known coronary disease (4, 14, 15). Whilst neither of these medications carry the antiplatelet effect of Aspirin, they do have important anti-arrhythmic and plaque stabilizing effects. The use of either of these medications in combination with aspirin potentially overestimates the relative risk benefit conferred by aspirin alone. Similarly, much of the evidence for aspirin's use in the post-surgical setting stems from trials conducted nearly 20 years ago. The multi-centre Perioperative Ischemia Group investigated the role of aspirin in reducing complications or death following cardiac surgery. They found that aspirin within 48 h post revascularization was associated with reduced rates of death and ischemia complications particularly stroke, with the results forming much of today's widespread prescribing to surgical patients (4). However, the impressive results of aspirin in these early studies were likely to again be in part a reflection of the population sample. The median age of patients receiving CABG was 64 with only 40% on an ACEI and 62% receiving a beta blocker (4). Hence the relative benefit of aspirin as part of contemporary best medical therapy is not clearly evidence based. In days where perioperative physicians and surgical optimization are common practice, the once uncommon and forgotten bleeding side effects of aspirin become more relevant when confronted with an ever-decreasing benefit with its use in the modern optimized patient.

Several studies have identified patient populations at higher risk of perioperative bleeding. These include patients who are older (16), have chronic kidney disease (17), a previous history of bleeding (18) or low body weight (19). Other predictors of bleeding after CABG include younger patients, racial minorities, non-elective surgery or those requiring haemodynamic support, history of cerebrovascular or peripheral vascular disease, chronic lung disease, more severe coronary disease and immunosuppressive therapy (20). There are also a number of large studies which have attempted to derive a risk score for predictors of bleeding for cardiac surgery (21–25). However these studies did not focus on predictors for elective CABG.

Evidence-based care depends on robust literature supporting therapeutic interventions, and areas where there is insufficient or conflicting information should be pursued aggressively by clinicians and the scientific community to improve the care for patients worldwide (26). Currently there is a lack of evidence based on applicable risk stratification and thus consensus guidelines to individualise the use of aspirin in elective CABG. Further research should be aimed at further evaluating this patient cohort in order to develop clinical aids that guide perioperative aspirin use.

## Data Availability

The original contributions presented in the study are included in the article/Supplementary Material, further inquiries can be directed to the corresponding author.
